# Depression and quality of life in cancer patients with and without pain: the role of pain beliefs

**DOI:** 10.1186/1471-2407-8-177

**Published:** 2008-06-21

**Authors:** Azadeh Tavoli, Ali Montazeri, Rasool Roshan, Zahra Tavoli, Mahdiyeh Melyani

**Affiliations:** 1 Cancer Research Centre, Cancer Institute, Tehran, Iran; 2 Iranian Institute for Health Sciences Research, Tehran, Iran; 3 Department of Psychology, Faculty of Humanity Studies, Shahed University, Tehran, Iran

## Abstract

**Background:**

Pain is said to be one of the most feared and distressing symptoms of cancer and one that disrupts all aspects of life. The purposes of this study were: 1) to compare depression and quality of life among Iranian cancer patients with and without pain; and 2) to determine the relationships between pain beliefs and depression and quality of life.

**Method:**

A consecutive sample of gastrointestinal cancer patients attending to Tehran Cancer Institute were entered into the study. Three standard instruments were used to measure quality of life (the EORTC QLQ-C30), depression (the HADS) and pain beliefs (the PBPI).

**Results:**

A total of 142 hospitalized gastrointestinal cancer patients, 98 with pain and 44 without pain were studied. The main findings of this study were that cancer patients with pain reported significantly lower levels of role functioning, emotional functioning and global quality of life. They also showed higher levels of depression than cancer patients who did not experience pain. Among patients with pain, higher scores on pain permanence and pain consistency were positively and significantly associated with higher depression. Also, higher scores on pain consistency were negatively and significantly associated with global quality of life.

**Conclusion:**

This study has demonstrated the effect of cancer pain on patients' quality of life and emotional status and has supported the multidimensional notion of the cancer pain experience in cancer patients. Although these data are correlational, they provide additional support for a biopsychosocial model of chronic pain.

## Background

Pain is said to be one of the most feared and distressing symptoms of cancer [1,2] and one that disrupts all aspects of life [3]. Research has demonstrated that despite the effective analgesic therapy and an array of treatment modalities currently available as a result of advances in the area of neuro-oncology [4], there has not been a significant reduction in the prevalence of pain in patients with cancer [5].

Cancer pain has a significant impact on the overall quality of a cancer patient's life by influencing physical, psychological, and spiritual aspects [6]. Pain is the end product of a complex process that may involve emotional, spiritual, cognitive, and sensory components [7]. Cancer pain has characteristics of both chronic and acute pain. Like acute pain, cancer pain is directly associated with tissue damage. When cancer pain persists and worsens, it can serve as a sign of the progression of disease [8] and can create a sense of hopelessness because patients fear that their lives are not worth continuing or patients lose the meaning of living if they must live in pain [9].

During the past two decades, a small body of research has accumulated that suggests a relationship between pain and mood disturbance in patients with cancer. Gerbershagen et a1. [10] studied health-related quality of life inpatients with prostate cancer patients with and without pain. They found that depressive symptoms are significantly more frequent in pain patients than in patient without pain. Another study compared patients with and without pain who were matched by site and progression of disease [8]. Patients with pain scored higher on measures of depression as well as anxiety, hostility, and somatization.

Pain is a multidimensional experience, far beyond a nociceptive signal. It is rooted in our socio-cultural context and belief system [11]. Beliefs about cause, control, duration, outcome and blame are especially important. Even Lame et al. [12] indicated that quality of life in chronic pain is more associated with beliefs about pain, than with pain intensity.

Although certain cognitions and beliefs may be adaptive and help patients to cope with the experience of pain, others may actually contribute to increased pain and affective distress. Identification of adaptive and maladaptive pain-related beliefs and cognitions might improve our understanding of individual responses to chronic pain and contribute to more effective treatment interventions [13]. Cognitive-behavioral models of chronic pain emphasize the importance of pain-related cognitions and beliefs in pain adjustment [14]. Pain beliefs serve the function of helping human beings to gain a stable understanding of the events that they have, are, or will be experiencing [15]. The belief that pain is understandable has been associated with better treatment compliance and use of adaptive coping strategies, while the belief that pain is mysterious has been associated with greater use of catastrophizing [16].

In chronic non-cancer pain, it is generally agreed that the meaning assigned to pain can play an important role in the experience of pain and in the response to treatment. Several studies have demonstrated the impact of pain cognition on patients' pain experience, disability, distress, non-adherence, and outcome of treatment [12,17,18]. For instance, in a study [17] after controlling for demographics, employment status and pain severity, pain beliefs and cognitions accounted for a significant amount of the variance in general activity, pain interference, and affective distress. The importance of beliefs about the meaning of symptoms, how they should be managed, ability to control pain, and worry about the future have been shown to be associated with psychological functioning, physical functioning, coping, and response to treatment [18]. However, up until the last decade, very few researchers addressed the cognitive dimension of pain in cancer patients. Given the high importance of pain belief in quality of life [16], it is reasonable to assess the impact that pain beliefs, may have on depression and health-related quality of life in cancer patients. Thus, this aimed: 1) to compare depression and quality of life among Iranian cancer patients with and without pain; and 2) to determine the relationships between pain beliefs and depression and quality of life.

## Methods

### Design and data collection

This study was conducted at a large teaching hospital (Imam Hospital) in Tehran, Iran from November 2005 to April 2006. The intention was to interview all gastrointestinal cancer inpatients attending the hospital for their treatments. To be included in the study patients had to be a) over the age of 18 years, b) diagnosed within the last 12 months, and c) conscious and able to communicate. Patients were classified into two groups: pain group and pain-free group based on the presence or absence of cancer related pain. A psychologist in a face-to-face interview administrated the questionnaires. In the first part of the investigation, depression and health-related quality of life was compared between cancer patients with and without pain. The second part of the study addressed the association between pain belief and depression and quality of life. Verbal consents obtained from all patients prior to interview. The Ethics Committee of the Tehran University of Medical Sciences approved the study.

### Measures

#### 1. Depression

Depression was evaluated by the Hospital Anxiety and Depression Scale (HADS). It is a 14-item questionnaire consisting of two subscales: anxiety and depression. Each item is rated on a four-point scale giving maximum scores of 21 for anxiety and depression. Scores of 11 or more on either subscale are considered to be a significant "case" of psychological morbidity, while scores of 8–10 represents "borderline", and 0–7 "normal" [19]. The psychometric properties of the Iranian version of the HADS are well documented [20].

#### 2. Quality of life

Quality of life was assessed using the European Organization for Research and Treatment of Cancer Quality of Life Questionnaire (EORTC QLQ-C30). This is a core cancer-specific questionnaire containing 30 items measuring functioning, global quality of life, and disease- and treatment related symptoms [21]. The validation study of the Iranian version of the EORTC QLQ-C30 showed that it is a reliable and valid measure of quality of life in cancer patients [22]. For the present analysis we only used the functioning and global quality of life scores where a higher score indicates a better condition.

#### 3. Pain beliefs

Pain beliefs was measured using the Pain Beliefs and Perceptions Inventory (PBPI). This is a 16-item scale that measures the extent of agreement or disagreement with certain beliefs about pain [23]. The instrument measures four dimensions of pain beliefs: (1) constancy, the belief that pain is constant; (2) permanence, the belief that pain is permanent; (3) self blame, the belief that one is to blame for one's pain, and (4) mystery, the belief that pain is confusing and mysterious. Respondents rated the statements on a Likert scale from completely agree to completely disagree. For each dimension scores range between -8 and 8. Higher scores on each dimension indicate more maladaptive beliefs and perceptions about pain. The validation study of the Iranian version of the PBPI proved that it is a reliable and valid measure of pain beliefs and perceptions. [24].

### Statistical analysis

Descriptive statistics were used to describe the sample characteristics in terms of demographic and disease-related variables. The t-test was employed to determine if scores are differed in response to patients' experiences of pain. Among patients experiencing cancer-related pain, Pearson's correlation (r) was used to explore the relationship among pain-related beliefs, depression and global quality of life. A p value < 0.05 was regarded to be statistically significant.

## Results

### Patients' characteristics

A total of 98 cancer patients with pain and 44 cancer patients without pain (n = 142) participated in the study. Patients in the pain and pain-free groups were similar in their demographics and clinical characteristics. The detailed demographic and clinical data are summarized in Table 1.

**Table 1:** Demographic and clinical characteristics of the study sample

	**Patients with pain (n = 98)**	**Patients without pain (n = 44)**	**P value**
	**No. (%)**	**No. (%)**	

**Age (mean, SD)**	54.2 (14.2)	54.6 (14.2)	0.82
**Education (mean, SD)**	3.3 (4.4)	4.5 (5.7)	0.21
**Sex**			0.57
Male	53 (54.1)	26 (59.1)	
Female	45 (45.9)	18 (40.9)	
**Marital status**			0.1
Married	84 (85.7)	38 (86.4)	
Single	7 (7.1)	6 (13.6)	
Widowed	7 (7.1)	0 (0)	
**Cancer site**			0.18
Esophagus	29 (29.6)	12 (27.3)	
Stomach	29 (29.6)	13 (29.5)	
Small intestine	5 (5.1)	0 (0)	
Colon	17 (17.3)	14 (31.8)	
Rectum	18 (18.4)	5 (11.4)	

### Functioning, global quality of life, and depression

Functioning, global quality of life and depression scores for cancer patients with and without pain are presented in Table 2.

**Table 2:** Functioning, global quality of life, depression, and pain beliefs scores in patients with pain and without pain

	**Patients with pain (n = 98) Mean (SD)**	**Patients without pain (n = 44) Mean (SD)**	**P**
Functioning and global quality of life scores*			
Physical functioning	78.6 (19.7)	82.7 (14.2)	0.21
Role functioning	62.9 (28.3)	81 (20.5)	< 0.001
Emotional functioning	60.6 (22.9)	74.6 (17.4)	< 0.001
Cognitive functioning	94.7 (14.1)	96.2 (12.3)	0.54
Social functioning	80.9 (23.2)	84.8 (16.4)	0.31
Global quality of life	57.9 (23.6)	72.3 (14.2)	< 0.001
Depression score**			
HADS-D	9.2 (3.97)	6.8 (3)	< 0.001
Pain beliefs scores***			
Pain permanence	-4.45 (2.5)	NA	
Self-blame	-1.33 (4.6)	NA	
Pain consistency	-2.71 (3.6)	NA	
Mysteriousness	1.92 (3.4)	NA	

*. Higher scores indicate a better condition.

** HADS-D: Depression score derived from the Hospital Anxiety and Depression Scale. Higher score indicates a greater symptom.

*** Higher scores indicate more maladaptive beliefs. NA: not applicable.

Comparison of functioning and global quality of life scores between cancer patients with and without pain indicated that those who experienced pain showed a significant lower degree of global quality of life (P < 0.0001), physical (P = 0.001), emotional (P = 0.014) and role functioning (P < 0.0001). In addition, the analysis showed that patients in the pain group had significantly higher levels of depressive symptoms than patients in the pain-free group (P < 0.0001).

For patients with pain (n = 98), the mean (SD) pain beliefs scores on the PBPI were: pain permanence: -4.45(2.5); self-blame: -1.33(4.6); pain consistency: -2.71(3.6); mysteriousness: 1.92(3.4).

### Relationships between pain-related beliefs, depression and global quality of life for Patients with Pain

Pain consistency was significantly correlated with depression (r = 0.32, P = 0.001) and global quality of life (r = -0.31, P = 0.002). Pain permanence was significantly correlated with depression (r = 0.3, *P *= 0.003), but not with global quality of life. In addition, there were no significant relationships between depression, quality of life and self-blame and mysteriousness (Table 3).

**Table 3:** Relationship of pain beliefs, depression and global quality of life (n = 98)

	Depression r (p)*	**Global quality of life r (p)**
**Pain permanence**	0.3 (0.003)	-0.12 (0.25)
**Self-blame**	0.13 (0.21)	-0.14 (0.17)
**Pain consistency**	0.32 (0.001)	-0.31 (0.002)
**Mysteriousness**	0.18 (0.07)	-0.09 (0.37)

* r = correlation coefficient, p = significant level.

## Discussion

The results of this study provide several important implications for understanding the impact of cancer pain on patients' quality of life and depression. The findings of this study support the multidimensional notion of the cancer pain experience [8] and demonstrate the effect of cancer pain on the psychological aspect of Iranian cancer patients' quality of life. There have been few studies that directly compare emotional status of cancer patients without pain to those with pain. In contrast to the physiological components of cancer pain, there has been little prior research on other aspects of cancer pain experiences, such as psychological or emotional distress. The results of this study showed that pain has deleterious effects on cancer patients' emotional status. In this study, patients with cancer pain had significantly lower role, emotional functioning and global quality of life scores than patients without cancer pain.

Depressive symptoms are significantly more frequent in pain patients than in patient without pain. These results from the current study conducted in Iran is consistent with prior studies conducted in other countries [10,25,26] where patients with cancer pain reported significantly higher levels of perceived emotional distress due to pain than did those without pain. Lin et al. [25] studying Taiwanese cancer patients with and without pain found that cancer patients with pain reported significantly lower levels of performance status and higher levels of total mood disturbance than did cancer patients who did not experience pain after controlling for sex, disease stage, and recruitment site. In a study of 200 American cancer patients who were experiencing pain and 169 cancer patients who were pain-free, Glover et al. [26] found that patients who experienced cancer pain scored significantly higher on anxiety, depression, anger, fatigue, confusion, and total mood disturbance. It is argued that pain might play a causal role in producing depression [27]. For patients with progressive life-threatening diseases, pain can add greatly to the debilitating effects of the disease and foster hopelessness and fear [28]. Cancer threatens patients' existence and cancer pain may cause suffering which leads to emotional distress for cancer patients [29].

The cognitive dimension of pain refers to the way patients think about their pain and what the pain means for them, in terms of thoughts, beliefs, attitudes, and self-efficacy expectations. The meaning patients ascribe to their pain may differ among different individuals [30]. Beliefs about pain are assumed to play an important role in the process of coping by influencing both the initiation of coping strategies and a person's level of adjustment. The way a patient copes with pain is influenced by the thoughts about their pain and what the pain means for them [31]. Although a considerable body of knowledge exists on the role of pain cognitions in non-cancer patients, only a few studies in cancer patients have shown that pain beliefs are associated with pain intensity. Arathuzik [32] found that cognitive and emotional factors appeared to play a central role in the response to pain and in the coping methods used to deal with pain. Also it was found that a cognitive-behavioral intervention can change the ability to decrease pain [33]. Zimmerman et al. [34] found a relationship between pain intensity and psychological status. In general, the cognitive dimension of cancer pain received less attention compared to other aspects of cancer pain such as physical pain.

The results of the present study replicate and extend previous findings about relationship between pain beliefs and emotional functioning. In this study higher score on some pain beliefs and perceptions including pain permanence and pain consistency were positively and significantly associated with higher depression. Also, higher scores on pain consistency were negatively and significantly associated with global quality of life. These are similar to findings from previous studies where it was found that pain beliefs and cognitions were related to pain adjustment [17,24,35]. Asghari et al [24] studied the impact of pain-related beliefs in the adjustment to cancer pain. They asserted that higher scores on pain beliefs in cancer patients are positively and significantly associated with more severe pain and higher levels of pain interference. Also examining the effects of pain beliefs in cancer patients demonstrated that perceived control over pain had a direct effect on symptom distress and mediated the effect of beliefs about pain and pain level on symptom distress. Patients' perceived control over pain maybe an important component in pain management [35].

Although our findings should not be interpreted as indicating that negative beliefs cause mood disturbance, they do indicate the need for further research examining this relationship. It is likely that there might be a mutual relation among these variables. Greater use of longitudinal designs should help in understanding the ability of beliefs and cognitions to predict quality of life in patients who suffer from cancer related pain. However, the results of this study should be read with caution. This was a descriptive design and it would be useful to replicate this study using measures obtained by other methods, especially behavioral observations.

## Conclusion

The findings demonstrated that Iranian cancer patients, similar to cancer patients in other countries, are affected by cancer pain in many dimensions of their lives. The similarity in these patients' responses indicates that the negative impact of cancer pain is not culture specific. It is important for clinicians to make every effort to prevent cancer pain and to relieve pain effectively and promptly. Based on experiences from Western countries, pain therapy that addresses only one component of the pain experience might be destined to fail [36]. Interventions that address the multidimensional aspect of pain by relieving the patient's physical burden, psychological disturbance, and emotional distress are more likely to lead to long-term benefits, not only for patients in Western countries but also for Iranian patients. Our findings also have implications for treatment by providing an explicit rationale for targeting the reduction of maladaptive beliefs.

## Competing interests

The authors declare that they have no competing interests.

## Authors' contributions

AT was the main investigator and wrote the first draft of the manuscript. AM supervised the study, analyzed the data and wrote the final draft of the manuscript. RR supervised the study. ZT contributed to the study design. MM contributed to the data collection. All authors read and approved the final manuscript.

## Pre-publication history

The pre-publication history for this paper can be accessed here:


